# YSMR: a video tracking and analysis program for bacterial motility

**DOI:** 10.1186/s12859-020-3495-9

**Published:** 2020-04-29

**Authors:** Julian Schwanbeck, Ines Oehmig, Jerôme Dretzke, Andreas E. Zautner, Uwe Groß, Wolfgang Bohne

**Affiliations:** 1 Institute for Medical Microbiology, University Medical Center Göttingen, Göttingen, Germany; 2 Institute of Applied Mathematics, Leibniz University Hannover, Hannover, Germany

**Keywords:** Bacterial motility, Video tracking, openCV, Python, Video microscopy, 2D object tracking, Open-source software, Multi-object tracking

## Abstract

**Background:**

Motility in bacteria forms the basis for taxis and is in some pathogenic bacteria important for virulence. Video tracking of motile bacteria allows the monitoring of bacterial swimming behaviour and taxis on the level of individual cells, which is a prerequisite to study the underlying molecular mechanisms.

**Results:**

The open-source python program YSMR (Your Software for Motility Recognition) was designed to simultaneously track a large number of bacterial cells on standard computers from video files in various formats. In order to cope with the high number of tracked objects, we use a simple detection and tracking approach based on grey-value and position, followed by stringent selection against suspicious data points. The generated data can be used for statistical analyses either directly with YSMR or with external programs.

**Conclusion:**

In contrast to existing video tracking software, which either requires expensive computer hardware or only tracks a limited number of bacteria for a few seconds, YSMR is an open-source program which allows the 2-D tracking of several hundred objects over at least 5 minutes on standard computer hardware.

The code is freely available at https://github.com/schwanbeck/YSMR

## Background

Bacteria developed different types of motility, most of them driven by flagella or pili. The molecular processes that regulate motility in bacteria are an active area in research, as they form the basis for dispersion, tactile processes, and virulence in some pathogenic bacteria [1].

The particular type of bacterial swimming varies significantly among bacterial species and depends on the number of flagella and their distribution on the bacterial cell body [2]. The best studied example is the “run and tumble” motility type of *Escherichia coli*, in which counter clockwise rotation of flagella leads to a run phase, while clockwise rotation leads to a tumbling phase with a random cell rotation [3]. However, in recent years additional motility types were discovered, as the “forward-revers-flick” motility type in *Vibrio alginolyticus* [4] or the “stop-and-coil” type in *Rhodobacter sphaeroides* [5].

To study bacterial motility pattern, microscopic monitoring and analysis of single cell motility is required. Manual analysis of motility videos increases the risk of inadvertent cherry picking, as well as being tedious. The application of video tracking software is thus advisable for the quantification of various motility parameters such as the average speed, the length of travel paths, the time of swimming and tumbling, arc-chord ratio, percentage of immotile cells, and preferred direction of travel.

To our knowledge, video tracking software, which can be used for this purpose, have been designed with a high priority on tracking accuracy, as for example TrackMate2 [6], or require additional licences to be used [7].

However, a high accuracy tracking program monitors several parameters per tracked object per frame and can quickly run into hardware limitations, when several hundred objects are simultaneously analysed. Using such programs forces the user to choose between fewer cells per frame or shorter videos in order to be still functional.

We therefore felt the need for an open-source tracking program, which uses only simple parameters for tracking, in order to be able to cope with the large amount of tracked objects for at least 5 minutes. Here, we use the grey value of the bacterium for detection, and the distance between frames for tracking. As the sample size is very large, typically in the range of several hundred bacteria per frame, the loss of tracked objects is less important. After initial tracking, we subsequently filter out questionable data points and tracks in multiple steps. We also include the possibility for statistical analysis of the generated data.

### Software

#### File requirements

During the initial setup, YSMR requires various parameters to be set in an automatically generated settings file, “tracking.ini”. The file was created with the idea in mind that it should be simple to set the basic values, but still allow for more in-depth configurations. Basic required settings are pixel per micrometre factor, frames per second, frame dimensions, whether the bacteria are brighter or darker than the background, as well as whether rod shaped or coccoid bacteria are tracked.

In order to take advantage from multi-core CPUs, YSMR is designed to handle a video file per available processor core in parallel. Files can be loaded by specifying them in the file dialog, as arguments for the YSMR function, or by specifying the path in the tracking.ini file. The user has to provide a video file in any format accepted by ffmpeg. We so far successfully tested .wmv, .avi, .mov, .mp4, and .mkv.

#### Bacteria detection by grey value

**Fig. 1:**
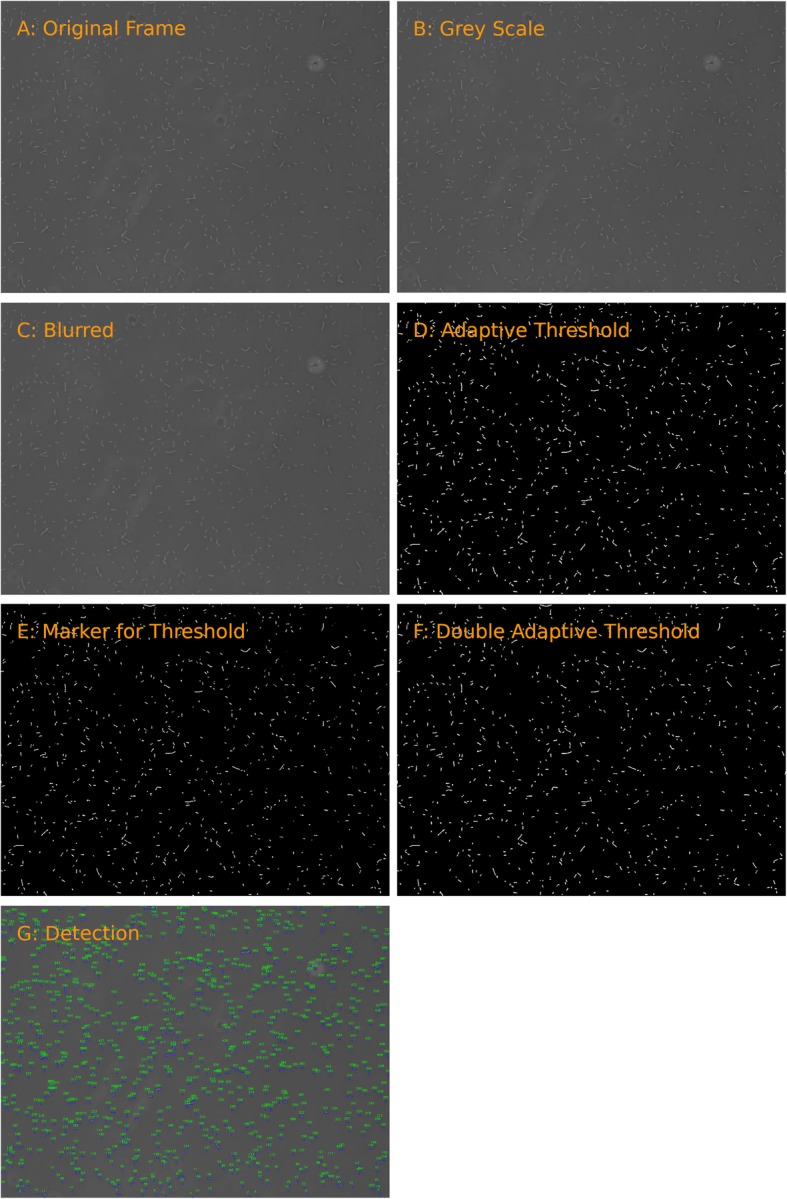
From original frame to final detection. **a**: the original frame from the video file. **b**: the frame is converted to grey scale. **c**: a Gaussian blur with a 3 by 3 kernel is applied. **d**: the adaptive threshold is applied, leaving white areas as potential bacteria. **e**: a second, higher, adaptive threshold is applied to generate markers. **f**: white areas from **d** which contain markers from E are used as outlines. **g**: each area is encased in a rectangle and assigned a unique ID, displayed on the original frame

Recorded bacteria can be either brighter or darker than the background. The program reads one frame at a time. During the process of bacterial detection, the frame is first converted into grey-scale (see Fig. 1 a & b). The noise in the frame is reduced by a 2D Gaussian blur in order to reduce the rate of false positive areas (Fig. 1 c). are set using an adaptive threshold with an 11 by 11 Gaussian kernel (Fig. 1 d). In order to exclude erroneous detections, a second adaptive Gaussian threshold with an increased threshold is used to generate marker positions (Fig. 1 e). Whenever no white area from Fig. 1 e is contained within a white area from Fig. 1 d, that area is disregarded. From this, the frame depicted in Fig. 1 f is generated. The outermost points of the edges in Fig. 1 f are then used to create a rectangle and each newly generated rectangle receives a unique ID (Fig. 1 g). The centre-point of the rectangle (named “tracking target”) is set as the x-, y-coordinate of the bacterium, which is used for tracking.

Optionally, either simple adaptive thresholding, without the marker based approach, or simple thresholding based on the grey value of the frame can be used. For simple thresholding, the average grey value of the entire frame, its standard deviation, as well as a user definable offset is used to calculate a threshold value. To reduce fluctuations in the threshold, a moving average over a timeframe of 5 s is used. Using the previously calculated threshold value, the frame is converted to a binary black and white image in which bacteria are always defined as white areas which are subsequently used for edge detection.

#### The tracking process

Tracking is performed by calculating the distances of all tracking targets between each frame and joining the neighbours with the least distance between frames. In addition, a Gaussian sum finite impulse response filter using a constant velocity model is used to filter the measured signal as well as to produce predictions of positions for tracking [8]. When a new frame has more tracking targets than a previous frame, new IDs are assigned to the additional tracking targets. Tracking targets which cannot be assigned will be given the width and height dimensions of 0.0 for missing frames, while their last predicted x-, y-coordinate will be used for tracking. Centre-points that cannot be assigned within 1 second of the last detection will be removed. The generated information per frame, consisting of ID, filtered position, width, height, time, and rotation angle, is periodically saved to disc in a .csv file. When all frames have been read, the resulting .csv file is sorted by ID and time.

#### Track selection

The data is then loaded as a pandas data frame and all tracks are checked for plausibility or errors before further calculations are performed. Track selection is performed in two parts, as some erroneous data points as well as entirely too short tracks can be excluded directly, which saves processing time and increases precision in the secondary finer selection step.

(i) In the initial step, entire tracks are discarded. This includes all tracks whose bounding rectangles are on average below or above user specified bacterial size limits. The lower limit discards most events which have a small bounding rectangle for a few frames, followed by frames with an area of zero for 1 second, averaging close to zero in total. The upper limit excludes areas of bright spots caused by dirt, chromatic aberrations in the microscope, or other unwanted objects. We find that using a lower limit of 20% of the average bacterial size in px^2^ and an upper limit of five times that area can be used as a rule of thumb for the initial broad exclusion limits. When a user defined minimum tracking time is given, all tracks below this limit at this stage are also excluded.

(ii) In the second step, erroneous single measurements are deleted. This includes all single measurement points where the area is zero. This is caused by the tracker, which zeros width and height when the track cannot be linked in between frames. The position given at such points is the last predicted position.

(iii) Optionally, single data points can be excluded if they surpass a specified multiple of the average bounding rectangle area of a track. This can occur when bacteria overlap, which increases the size of the bounding rectangle by the area of the second bacterium. In this case, one bacterium is erroneously assigned a larger size, while the other cannot be tracked.

After removing erroneous data points and tracks, each track is sequentially filtered through user defined criteria. A count of how often a track has been excluded through each criterion is reported afterwards. We will hereafter refer to removed measurement points as “gaps” in the measurement. The following criteria are included: (iv) first, the duration in time of the track must be above a user defined minimum time limit, if one is specified. (v) The track may not have more than a set amount of consecutive frame gaps, which ensures continuous tracking. (vi) Optionally, distance outliers within tracks can be calculated. Outliers are defined as those above the outer fence of all distances. As this is prone to error when too many tracked objects are immotile, the feature can optionally disable itself if the detected outliers are above a specified percentage of all data points. If too large consecutive gaps in measurement or distance outliers are present, the track is split and both halves are analysed again, starting at the first check. (vii) The data points of the track may not have more than a user defined percentage of gaps. (viii) The average bacterial size within a track can be used as an exclusion parameter. If the average size is outside a percentile of all size measurements, the track is excluded. The percentile is also defined by the user. Area outliers missed by the hard limit of the initial sorting step ‘i’ are excluded at the possible expense of excluding a fraction of correct tracks. (ix) The average position of the track must not be within a given percentage of the screen edges. These tracks can be excluded as a precaution, since IDs near the screen edges could be wrongly reassigned when other bacteria enter the frame.

If all checks are passed, the track is added to the results. If a track was split and several parts pass the checks, the longest part is selected and all others are discarded. Optionally, tracks can be limited to a user defined maximal duration. When an upper time limit is set the track is shortened to the exact duration or, if a gap is at that position, the closest data point below the set time limit. This behaviour can be changed so that shortened tracks which fall below the limit are discarded instead.

### Data processing, analysis and illustration

The tracking process can either be displayed during analysis or saved as an .avi file encoded in MJPEG. The generated raw data from the tracking, the results from the subsequent fine selection, as well as the results from the statistical analysis can each be saved as an individual .csv file. The statistical analysis .csv files can also as a convenience function be collated into a .xlsx file.

Tracks can be graphically visualized in a coordinate graph with a marker at the starting position, or as a rose graph with starting x−/y-coordinates set to 0,0. In each case, the tracks are coloured depending on travelled distance.

Tracks can be analysed for the following parameter: (i) total travelled distance (μm), (ii) speed (μm/s), (iii) duration (s), (iv) maximum distance between tracked positions (μm), (v) percentage of time where bacterium was motile, (vi) turn points per second, and (vii) arc-chord ratio. Generated statistics can be displayed as violin plots.

### Comparison to TrackMate

TrackMate is a widely used tracking program designed for sub-pixel localisation and tracking of eukaryotic cells [6]. Smaller objects, such as bacteria, can also be tracked. It is in contrast to YSMR a semi-automatic application requiring user supervision and input during tracking. A further limitation is the maximum video length when tracking a large number of objects due to increasing RAM requirements. We compared the performance and results between YSMR and TrackMate on the example video which was shortened to 30 s (Supplementary Fig. 1, Supplementary Video 1, and Supplementary Table 1). The analysis was done in comparable time (YSMR 140.5 s, TrackMate 141.8 s) with default options for YSMR. However, YSMR used fewer computational resources, since it is designed for parallel analysis of multiple videos. YSMR used only one thread, whereas TrackMate used four. The peak RAM usage was 6.3 GB for TrackMate and 1.1 GB for YSMR. Comparing the results, when overlaid, YSMR detects 91.1% of all positions that overlap with those detected by TrackMate. Mean values for speed, distance, displacement, arc-chord ratio, and percentage of motility are in a similar range with variations between 6.23 and 19.81% of the means (Supplementary Table 1).

## Discussion and conclusion

YSMR is a python program for generating tracking data and statistics from video files depicting motile bacteria. It offers the possibility to determine and quantify the most relevant bacterial motility parameters, for example total travelled distance, speed, percentage of time where bacteria are motile and turn points per second, among others. A thorough analysis of these data can provide the basis for novel insights into the motility behaviour of bacteria and its regulation.

Existing tracking programs have a focus on the high accuracy of generated tracking trails from video files and were often performed on very small sample sizes, due to the need to track single cells frame-by-frame throughout the video. In contrast, YSMR is optimized for the simultaneous tracking of a very large number of bacteria, typically in the range of 100–1000 per frame from video files generated with a 10x objective. YSMR is based on a moderate fidelity, high selectivity approach, which keeps the processing time short. Instead of using computational intensive approaches for bacterial detection, for example machine learning, we found that the detection could be reduced to a very simple approach, namely finding bacteria by grey value threshold. We concluded that we could simplify the whole process down to the described detection and tracking mechanism, as long as in a secondary step we rigorously select against suspect data points. The entire process is inexpensive enough to be run on standard desktop computers and was adapted to take advantage of multicore processors for parallelisation.

In our setup, efficient tracking of flagellated *Bacillus subtilis* was possible with YSMR by using video files generated with a 30 fps camera (Aptina CMOS Sensor 18MP 1/2.3″ Color) on a microscope with a 10x objective (Nikon Eclipse TE2000-S, Nikon PlanFluor 10x). The comparatively large depth of the focal plane of a 10x objective minimizes the number of bacteria that move out of focus during tracking. A frequent experimental design is to analyse bacterial motility statistics for a population under varying conditions (for example different pH, nutrient availability, growth phase, or cell densities). YSMR can analyse generated video files for this purposes quickly and in parallel. On the other hand, if the exact motility pattern of single bacteria, the movement in 3D, or bacteria-bacteria interaction is of interest, or the cell density is below 50 objects per frame, other programs with higher tracking accuracy might be a better choice. To assess the performance of YSMR for a given task, the debug option lets the user review the detection process. For a straightforward assessment of the generated data, the save video option displays the generated measurements directly on the original video. YSMR was optimized to analyse bacterial motility statistics from a large number of bacteria in short time. Even if more precision is required, YSMR can still be used as a simple and quick pre-screening in order to select files for more complex and time consuming processes.

### Availability and requirements

Project name: YSMR v 0.1.0.

Project home page: https://github.com/schwanbeck/YSMR

Operating systems: Platform independent.

Programming Language: Python ≥3.6.

Other requirements: opencv (opencv-contrib-python/opencv-python v3/v4, v2 untested), numpy, pandas, tkinter, matplotlib, scipy, seaborn.

Optional: xlsxwriter.

License: GNU GPL v3.0.

Any restrictions to use by non-academics: None.

## Supplementary information

**Additional file 1: Supplementary Video 1.** A video of motile *Bacillus subtilis* 168 in LB. The video was taken with a 30 fps camera (Aptina CMOS Sensor 18MP 1/2.3″ Color) on a microscope with a 10x objective (Nikon Eclipse TE2000-S, Nikon PlanFluor 10x).

**Additional file 2: Supplementary Figure S1.** Comparison between tracked positions of YSMR and TrackMate. All x- and y-axes are in pixel. The example Video (supplementary Video 1) was truncated to 30 s, as TrackMate cannot analyse the full video. Each frame was converted to 8 bit grey scale .tiff files. The images were loaded into Fiji and subsequently tracked with TrackMate. We applied no filters to the results generated by TrackMate. The first 10 s of the results were overlaid with those generated with YSMR for the same section of the video in **supplementary Figure 1 A.** The position of five randomly picked tracks, which were compared in detail between TrackMate and YSMR (Fig. 1 B-F) are marked with blue arrows. Spots in orange were only identified by YSMR (2.87% of all spots). Spots in black were only identified by TrackMate (67.78% of all spots). Spots in green were identified by both (29.35% of all spots). **Supplementary Figure 1 B-F.** show individual tracks in direct comparison with YSMR in orange and TrackMate in black. The majority of tracks which were only recognised by TrackMate and not by YSMR are located in the periphery. For YSMR the standard settings for track selection were used, which actively removes tracks which are mainly near the frame edges in addition to otherwise questionable tracks, leaving only high quality tracks. This explains the lower number of tracks recognised by YSMR compared to TrackMate in this example.

**Additional file 3: Supplementary Table S1.** A comparison between results generated by TrackMate and YSMR. All tracks with a duration between 5 and 10 s were selected and the mean of motility parameter was calculated.

## Data Availability

An example video of *Bacillus subtilis* is provided in the supplementary.

## Abbreviations

ACR: Arc-chord ratio
CPU: Central processing unit
FIR: Finite impulse response filter
GB: Gigabyte
GSFF: Gaussian-sum finite impulse response filter
ID: Identifier
IQR: Interquartile range
LSFF: Least square finite impulse response filter
MJPEG: Motion JPEG
OS: Operating system
YSMR: Your Software for Motility Recognition
csv: Comma-separated values file
fps: Frames per second
pH: Power of hydrogen, negative base 10 logarithm of the concentration of hydrogen
px: Pixel
