# Supplementary figures and images for: YSMR: a video tracking and analysis program for bacterial motility
Source: BMC Bioinformatics. 2020 Apr 29;21:166. doi: 10.1186/s12859-020-3495-9 (PMC7191716; doi:10.1186/s12859-020-3495-9)

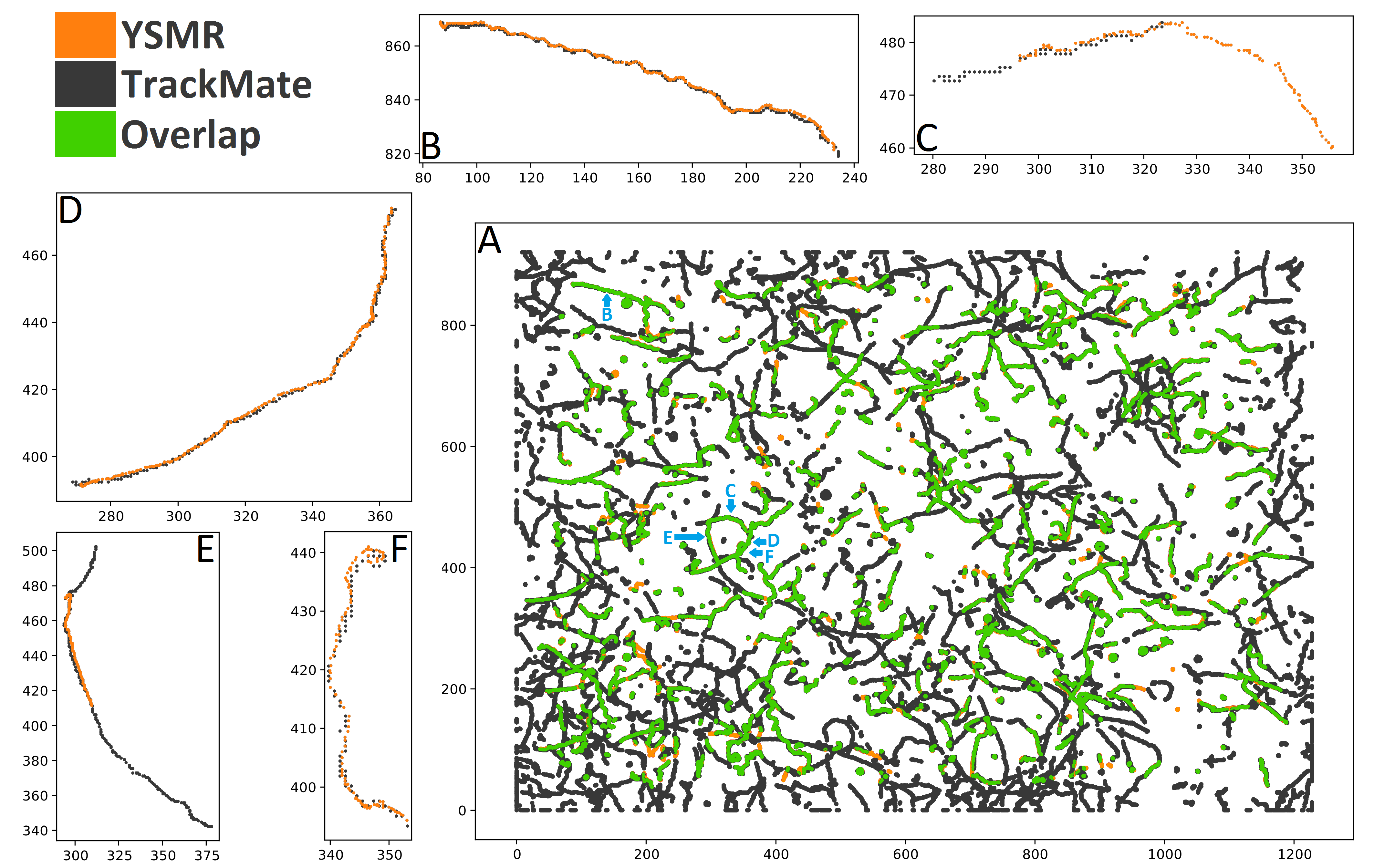

Supplement: Supplementary file 2 — Additional file 2: Supplementary Figure S1. Comparison between tracked positions of YSMR and TrackMate. All x- and y-axes are in pixel. The example Video (supplementary Video 1) was truncated to 30 s, as TrackMate cannot analyse the full video. Each frame was converted to 8 bit grey scale .tiff files. The images were loaded into Fiji and subsequently tracked with TrackMate. We applied no filters to the results generated by TrackMate. The first 10 s of the results were overlaid with those generated with YSMR for the same section of the video in supplementary Figure 1 A. The position of five randomly picked tracks, which were compared in detail between TrackMate and YSMR (Fig. 1 B-F) are marked with blue arrows. Spots in orange were only identified by YSMR (2.87% of all spots). Spots in black were only identified by TrackMate (67.78% of all spots). Spots in green were identified by both (29.35% of all spots). Supplementary Figure 1 B-F. show individual tracks in direct comparison with YSMR in orange and TrackMate in black. The majority of tracks which were only recognised by TrackMate and not by YSMR are located in the periphery. For YSMR the standard settings for track selection were used, which actively removes tracks which are mainly near the frame edges in addition to otherwise questionable tracks, leaving only high quality tracks. This explains the lower number of tracks recognised by YSMR compared to TrackMate in this example. [file 12859_2020_3495_MOESM2_ESM.png]
